# Macroporous Polymer–Protein Hybrid Materials for Antibody Purification by Combination of Reactive Gelation and Click-Chemistry

**DOI:** 10.3390/ma12101580

**Published:** 2019-05-14

**Authors:** Marcel Lorenz, Carolina Paganini, Giuseppe Storti, Massimo Morbidelli

**Affiliations:** Department of Chemistry and Applied Biosciences, Institute of Chemical and Bioengineering, ETH Zurich, 8093 Zurich, Switzerland; marcel.lorenz@chem.ethz.ch (M.L.); carolina.paganini@chem.ethz.ch (C.P.); giuseppe.storti@chem.ethz.ch (G.S.)

**Keywords:** macroporous Materials, click-chemistry, reactive gelation, protein immobilization, antibody purification

## Abstract

Clickable core-shell nanoparticles based on poly(styrene-*co-*divinylbenzene-*co-*vinylbenzylazide) have been synthesized via emulsion polymerization. The 38 nm sized particles have been swollen by divinyl benzene (DVB) and 2,2’-azobis(2-methylpropionitrile) (AIBN) and subsequently processed under high shear rates in a Z-shaped microchannel giving macroporous microclusters (100 µm), through the reactive gelation process. The obtained clusters were post-functionalized by “click-chemistry” with propargyl-PEG-NHS-ester and propargylglicidyl ether, yielding epoxide or NHS-ester activated polymer supports for bioconjugation. Macroporous affinity materials for antibody capturing were produced by immobilizing recombinant *Staphylococcus aureus* protein A on the polymeric support. Coupling chemistry exploiting thiol-epoxide ring-opening reactions with cysteine-containing protein A revealed up to three times higher binding capacities compared to the protein without cysteine. Despite the lower binding capacities compared to commercial affinity phases, the produced polymer–protein hybrids can serve as stationary phases for immunoglobulin affinity chromatography as the materials revealed superior intra-particle mass transports.

## 1. Introduction

With the pioneering work of Davis and coworkers in the 1970’s who attached polyethylene glycol (PEG) to proteins, a technique referred to as pegylation, the first polymer-protein conjugates were formed [1]. Ever since, biomolecule–polymer hybrids have been thoroughly investigated as they combine the advantages of synthetic polymers such as mechanical properties and convenient processability with the inherent specificity of proteins in biological systems, making these hybrids useful as therapeutic agents, molecular sensors or switches [2,3,4]. Altogether, polymer bioconjugates have been used for various applications in biomedicine [5,6], biotechnology [7,8], and nanotechnology [9,10] for instance as switches to control protein activity [11], as molecular sensors [12] or for affinity chromatography to purify immunoglobulins from human plasma [13]. 

Thereby, different pathways by which proteins can be attached to a polymer support have been described and commonly involve physical adsorption, specific biological recognition, self-assembly, or covalent immobilization [14]. The covalent attachment is usually preferred whenever protein leaching from the support is a major concern. To attach proteins to a polymeric support covalently, the polymer of choice must be modified towards protein-reactive end-groups that facilitate coupling between the polymer and the amino acids in the proteins side chain. Among others, *N*-hydroxysuccinimide (NHS) esters are highly activated to nucleophilic addition and are frequently used for protein immobilization onto polymers. However, the disadvantage of this approach is that heterogeneous bioconjugation often occurs due to non-specific coupling with multiple and random amine residues resulting in a significant reduction in bioactivity [15]. Site-specific modifications of amines have been attempted by exploiting the lower p*K*_a_ of the N-terminal α-amine. However, even if the coupling reaction is conducted under slightly acidic conditions, heterogeneity is often still observed and unpredictable conjugation of multiple chains can take place. The latter can be detrimental to the properties of the biomacromolecule, potentially resulting in reduced enzymatic or receptor-binding activity and/or toxicity [16,17,18]. Besides random coupling, also the limited hydrolytic stability is a potential concern for reactions taking place in aqueous media, which hampers the usage of NHS-esters for specific applications [19]. Therefore, other approaches that result in well-defined conjugates have also been explored. One example is the exploitation of thiol-reactive polymers. The sulfhydryl group of cysteine can be employed for such purposes as cysteine is a scarcely occurring amino acid making site-specific bioconjugation feasible [20]. Another approach towards a fully biorthogonal coupling reaction would be the introduction of non-canonical amino acids bearing for instance alkyne or azide functionality to undergo 1,3-dipolar cycloadditon [21], which is conducted using a Cu(I) catalyst and commonly referred as click-chemistry [22]. Catalyst-free [3 + 2] azide-alkyne cycloadditions for covalent modifications of biomolecules have been described exploiting the ring-strain of cyclooctynes which enable in-vivo applications [23].

**Figure 1:**
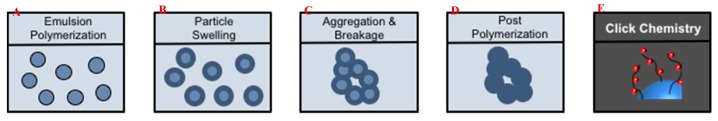
Sketch of the reactive gelation process towards functionalized macroporous polymeric scaffolds. (**A**) core-shell nanoparticle synthesis by emulsion polymerization; (**B**) swelling of the softer shell with crosslinker divinyl benzene (DVB) and initiator 2,2’-azobis(2-methylpropionitrile) (AIBN); (**C**) aggregation by applying high shear rates; (**D**) post-polymerization at 70 °C; (**E**) surface modification by click chemistry.

In this work we present a solvent-free process, called reactive gelation [24], to produce (macro)porous polymer scaffolds bearing azide groups on the surface as shown in our previous work [25]. As schematically shown in Figure 1 (first four boxes), the reactive gelation process contains four main steps: the synthesis of polymeric core-shell nanoparticles, their swelling with cross-linker and lipophilic initiator, high-shear aggregation to form porous microclusters, and the post-polymerization step to harden the obtained fractal materials. Each step is fully separated from the others, and allows better control of the final product properties as discussed earlier [26,27,28,29,30,31]. By exploiting the concept of copper-mediated alkyne-azide cycloaddition (CuAAC), the produced azide-containing polymers can readily be modified chemically yielding activated polymer templates for protein immobilization upon introducing NHS-ester or epoxides to the surface (Figure 1, last box). As a model protein, the high-affinity ligand Staphylococcus aureus protein A (SPA), the gold standard in the downstream processing of monoclonal antibodies, has been attached in two different ways: (i) via multipoint attachment of free primary amine groups in the protein backbone (in form of lysine), and (ii) by single point attachment with the recombinant protein A ligand that additionally contains a cysteine unit at its C-terminus, allowing a thiol-epoxide ring opening reaction. It is noteworthy that affinity chromatography with protein A immobilized polymer beads is usually the first step to separate desired monoclonal antibodies from cell and process-related impurities. However, commercial protein A beads commonly exhibit only small pores (~150 nm), resulting in a diffusion limited mass transport throughout the packed chromatography columns. Therefore, the purification process becomes time-consuming and vastly expensive, which could be resolved by using materials with larger pores. Since the herein presented microclusters possess pore sizes in the micrometer range, they allow a convective flow and thus a chromatographic performance that is independent of the applied process rate.

## 2. Materials and Methods 

### 2.1. Chemicals

*4-*vinylbenzyl chloride (Sigma Aldrich, Buchs, Switzerland), DMF (VWR, Schlieren, Switzerland), sodium azide (Sigma Aldrich), diethyl ether (Sigma Aldrich), MgSO_4_ (Sigma Aldrich), sodium dodecyl sulfate (Sigma Aldrich), propargyl glicidyl ether (Sigma Aldrich), propargyl-dPEG1-NHS–ester (Rapp Polymer, Tübingen, Germany), sodium ascorbate (Sigma Aldrich), Protein A (Syd Labs, Natick, MA, USA), Cys-Terminated protein A (ProspecBio, East Brunswick, NJ, USA), styrene (Sigma Aldrich), divinyl benzene (Sigma Aldrich), potassium persulfate (Sigma Aldrich), 2,2’-azobis(2-methylpropionitrile) (AIBN) (Sigma Aldrich), copper sulfate (Sigma Aldrich), sodium ascorbate (Sigma Aldrich), ethylenediaminetetraacetic acid (Sigma Aldrich), sodium phosphate (Sigma Aldrich), sodium sulfate (Sigma Aldrich), potassium bromide (Sigma Aldrich), ethanol amine (Sigma Aldrich), citric acid (Sigma Aldrich), sodium chloride (VWR).

### 2.2. Synthesis of 4-Vinylbenzyl Azide (VBA)

VBA was synthesized as reported previously [32]. To a solution of 4-vinylbenzyl chloride (20.2 g, 132.4 mmol) in DMF (300 mL) was added sodium azide (17.2 g, 264.8 mmol). The suspension was stirred for 48 h at room temperature before diluting it with 300 mL water. The product was extracted with diethyl ether (3 × 300 mL). The organic phase was washed with brine (3 × 200 mL) and dried over MgSO_4_ before evaporating the solvent under reduced pressure. The product (15.9 g, 100 mmol, 76%) was obtained as an orange oil. ^1^H-NMR (300 MHz, CDCl_3_) δ/ppm = 7.45–7.42 (m, 2 H, ArH), 7.30–7.27 (m, 2 H, ArH), 6.73 (dd, 1 H), 5.77 (dd, 1 H) 5.29(dd,1 H), 4.32 (s, 2H). 

### 2.3. Latex Synthesis

The primary particles are produced via a semi-batch emulsion polymerization protocol. In the first phase, (core synthesis) a hard and highly crosslinked seed of nanoparticles made of 80 wt % styrene (St) and 20 wt % divinyl benzene (DVB) is produced. In a 1-L round bottom flast, 650 g water and 4.1 g SDS were charged and heated to 70 °C. Afterwards, 1.5 g potassium persulfate (KPS), dissolved in 100 g water is injected (initiator shot1, IS1), and directly followed by adding the monomer feed containing 45.0 g water, 32.0 g styrene, 8.0 g DVB and 1.3, g SDS. In order to guarantee starved polymerization conditions, the monomer mixture is dropped to the system slowly with 0.5 mL min^−1^ using a Hitachi L-7110 HPLC pump (charged feed 1, CF1, Hitachi, Tokyo, Japan). During the polymerization process the particle size was constantly monitored by dynamic light scattering until the particles achieved a diameter of 30 nm. Afterwards, the shell synthesis was started. The shell is much softer, and only slightly crosslinked and comprises *4*-vinylbenzyl azide (VBA). The shell usually contains 2% DVB, 39% VBA, and 59% styrene. The core and shell syntheses are performed sequentially and under monomer-starved polymerization conditions, thus directly feeding the mixture required for the shell after the core synthesis. The shell feed contained 17.3 g styrene, 0.6 g DVB and 11.3 g VBA and was added to the system with 0.25 mL min^−1^. When the monomer feed was changed to the shell feed, another initiator show containing 0.3 g KPS dissolved in 12 g water was added to the system. The shell feed was stopped once the final particle size achieved 38 nm. An overview of the used chemicals and quantities is given in Table 1.

### 2.4. Latex Swelling

The obtained latex is diluted with deionized water to a solid content of 8 wt %. Subsequently, divinyl benzene (20% with respect to solid polymer) and oil-soluble initiator (AIBN, 5% with respect to the added DVB) are added and stirred for 12 h. 

### 2.5. High-Shear Destabilization 

After the latex was swollen for 12 h (see Section 2.4.), it was aggregated using the high shear device HC-5000 (Microfluidics, Westwood, MA, USA) equipped with a L 30 Z microchannel (rectangular cross section of 5.76 × 10^−8^ m^2^, length of 5.8 mm). The generated shear rate $\dot{\gamma }$(1/s) is correlated to the pressure drop through the channel, ΔP, by the following empirical relationship: $\dot{\gamma }$ = 2.27 × 10^5^·ΔP^0.64^ where ΔP is the applied pressure drop in bar. In this work, aggregation was done using a ΔP of 120 bar, corresponding to a flow rate in the channel of 912 mL/min, a residence time of 0.94 ms, and a shear rate of 4.8 × 10^6^ s^−1^.

### 2.6. Synthesis of Cluster-Epoxy

300 mg azided microclusters, 100 mL dried DMF, 40 mg propargyl glycidyl ether, and 129 mg CuSO_4_ were charged in a 250 mL 3-neck-flask and sealed with a septum. Then, 401 mg sodium ascorbate were added to the solution under nitrogen counter stream. The solution was stirred for 3 days at room temperature (RT). Afterwards, the microclusters were exhaustively washed with EDTA solution, H_2_O, and brine prior resuspension in coupling buffer (0.1 M sodium phosphate, 0.5 M Na_2_SO_4_, pH 7.5). 

### 2.7. Synthesis of Cluster-NHS 

300 mg azided microclusters, 100 mL dried DMF, 30 mg propargyl-dPEG1-NHS ester and 125 mg CuSO_4_ were charged in a 250 mL round-bottom flask. The flask was sealed with a septum and the solution sonicated for 5 min in a in a VWR Ultrasonic Cleaner. Afterwards, 400 mg sodium ascorbate were quickly added to the solution and the mixture was stirred at 450 rpm for 3 days at RT under nitrogen counter stream. Afterwards, the microclusters were exhaustively washed with EDTA solution, H_2_O, and brine prior resuspension in coupling buffer (0.1 M sodium phosphate, 0.5 M Na_2_SO_4_, pH 7.5). 

### 2.8. Protein A Immobilization

The NHS or epoxide polymer clusters were transferred to a solution of protein A at a concentration of 10 mg/mL in 15 mL coupling buffer (0.1 M sodium phosphate, 0.5 M Na_2_SO_4_, pH 7.5). The solution was stirred at 300 rpm for 4 h at RT. After reaction with protein A, the mixture was resuspended in 1 M ethanolamine in coupling buffer and stirred at 300 rpm for 45 min at RT to block the unreacted NHS-esters and subsequently washed with deionized water.

### 2.9. Methods

Elemental analysis data were obtained from the Micro-Laboratory of ETH Zürich with the instrument Leco TruSpec Micro for C, H, N. The monomer conversion was determined from the solid content of the latex by using a HG 53 Moisture Analyzer from Mettler-Toledo. The particle size and the corresponding polydispersity index were measured by dynamic light scattering using a Zetasizer Nano ZS (Malvern Panalytical, Malvern, UK). The size of the obtained microclusters was determined by static light scattering (SLS) using a Mastersizer 2000. The click yield was calculated from FT-IR spectra comparing the ratios of the areas of the characteristic azide stretching band at 2100 cm^−1^ and the aromatic C–H stretching band between 2800–3100 cm^−1^. To do this, the sample (5 wt %) was mixed with KBr and analyzed using a Tensor 27 spectrometer (Bruker Optics, Billerica, MA, USA). Scanning electron microscopy was done using Leo 1530 SEM (Zeiss, Oberkochen, Germany) at a voltage of 5 kV equipped with a SE Inlens detector. The sample was coated with 4 nm platinum layer prior to the measurement. The pore size distribution was measured by mercury porosimetry using Pascal 140 and Pascal 440 (Thermo Scientific, Waltham, MA, USA). The BET measurement was done on a TriStar 3000 (Micromeritics, Unterschleissheim, Germany). 

### 2.10. Quantification of Immobilized Protein A and Static Binding Capacity

Approximately 15 mg of cluster-epoxy were charged in eppendorf tubes and subsequently loaded with a known amount of Cys-terminated protein A (0.025 mg, 0.05 mg, 0.075 mg, 0.1 mg, 0.125 mg, 0.15 mg, 0.175 mg, 0.2 mg, 0.25 mg, 0.3 mg, 0.35 mg, 0.4 mg, 0.75 mg) and 1.1 mL coupling buffer. Afterwards, the eppendorf tubes were gently shaken at RT for 24 h. Then, the microclusters were filtered and suspended in 1.0 mL 0.1 M ethanolamine solution and shaken at RT for another 24 h. Afterwards, tubes were centrifuged at 3500 rpm for 6 min and the solution was removed. The obtained microclusters were resuspended in 2 mL equilibration buffer (20 mM phosphate, 150 mM NaCl, pH 7.5). Next, the tubes were centrifuged at 3500 rpm for 6 min, the solution was removed and a solution of antibodies (4.3 mg/mL) was added and gently shaken at RT for 2 h. Then, the eppendorf tubes were centrifuged again and loaded with 2 mL equilibration buffer. This step was repeated once with equilibration buffer, twice with washing buffer (20 mM phosphate, 150 mM NaCl, pH 7.5) and again twice with equilibration buffer. After completion of washing steps, the tubes were centrifuged, the aqueous phase was removed and 0.5 mL elution buffer (50 mM citric acid, pH 3.0) were added and shaken at RT for 1 h. Lastly, after centrifugation, the eluate was passed through a 0.4 μL filter and neutralized with Tris buffer pH 8.0 (20% of total volume). IgG concentration in each eluate was determined by analytical Protein A chromatography with a Chromolyth Protein A 25-4.6 HPLC column, to evaluate the static binding capacity. The exact masses of solid microclusters in each eppendorf tube were determined by drying the eppendorf tubes at 40 °C under reduced pressure.

### 2.11. Breakthrough Curves & Dynamic Binding Capacity

All chromatographic runs were performed on a Contichrom® Lab-10 equipment (ChromaCon® AG) with wavelength of UV detection set to 280 nm. The preparative chromatography experiment was performed at 25 °C using and the ChromIQ® operating software. Conductivity, pH and UV at 305 nm were monitored online. The material was packed as a slurry in a Tricorn 5/50 column. All runs were performed using the same feed solution of pure IgG at a concentration of 4.3 mg/mL. The columns were loaded with 8 CV at 90 cm h^−1^. For all experiments, the dead volume of 0.3 mL between the UV detector and fractionator was taken into account and the dynamic binding capacities were calculated accordingly.The buffers used throughout this study for preparative protein A chromatography were: 100 mM Na-phosphate, pH 7.4 as binding and washing buffer; 20 mM phosphate, 1 M NaCl, pH 7.5 as washing buffer; 50 mM Na-citrate, pH 3.0 as elution buffer.

## 3. Results and Discussion

This study comprised the synthesis of azide-containing macroporous microclusters, their functionalization by click chemistry towards activated polymer scaffolds for protein immobilization, and a brief proof-of-concept of their use as stationary phases for the chromatographic purification of monoclonal antibodies.

### 3.1. Synthesis of Core-Shell Nanoparticles

This section deals with the first step of the reactive gelation protocol, the emulsion polymerization towards polymeric nanoparticles with core-shell architecture, exhibiting a hard, highly crosslinked, core on which a softer and less cross-linked shell is grown. Table 2 discloses the polymerization, which is carried out in a time frame of 6 h in total. The first three hours are dedicated to the core synthesis, revealing a low polydispersity index during the whole synthesis as the emulsion polymerization is done under monomer starved conditions. As one can see, the formed copolymer contains 80 wt % styrene (St) and 20 wt % divinyl-benzene (DVB). The high amount of used crosslinker DVB is crucial to achieve a very compact core that prevents approaching particles from coagulation. After three hours, the core had a particle size of 30 nm and a high conversion of 99%. Subsequently, the monomer feed was changed to start with the shell synthesis around the previously formed seed particles. The shell monomer mixture contained 59 wt % St, 2 wt % DVB and 39 wt % vinyl benzene azide (VBA) and was fed with 0.25 mL/min. When the total particle size of 38 nm, or the ratio in diameter for the core and the total particle size of 0.79, was achieved, the synthesis was stopped and a yellowish dispersion was obtained. The produced polymer contained 3.4 wt % nitrogen as revealed by elemental analysis (Table 3), which reflects an azide content of 0.3 mmol_azide_/g_polymer_.

### 3.2. Fabrication of (Macro)Porous Microclusters from Polymer Nanoparticles

After the particle synthesis, the attained dispersion was diluted to 8% and swollen with 20% DVB with respect to the solid polymer content and 5% 2,2′-Azobis(2-methylpropionitrile) (AIBN) with respect to the added DVB. The mixture was stirred for 12 h to guarantee a uniform distribution of initiator and crosslinker among the nanoparticles. The obtained nanoparticles are stabilized by electrostatic repulsion of surface-adsorbed SDS molecules, which bear negatively charged sulfate groups. Therefore, the aggregation was done by applying high shear rates (~4.8 × 10^6^ s^−1^) to overcome the energy barrier due to this electrostatic repulsion. After the aggregation step, clusters with fractal morphology were obtained and post-polymerized at 70 °C for another 12 h to harden the aggregates without affecting their internal morphology [29].

### 3.3. Characterization of Microclusters

**Figure 2:**
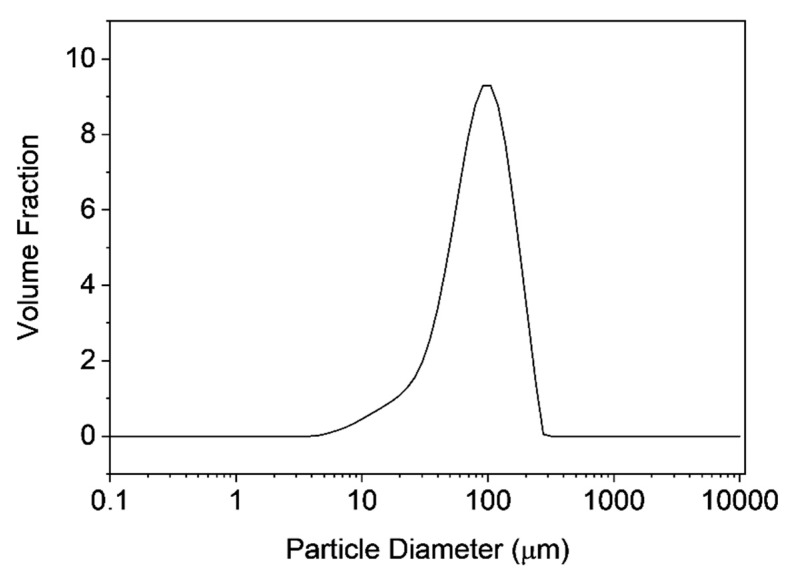
Particle size distribution of the microclusters measured by static light scattering.

**Figure 3:**
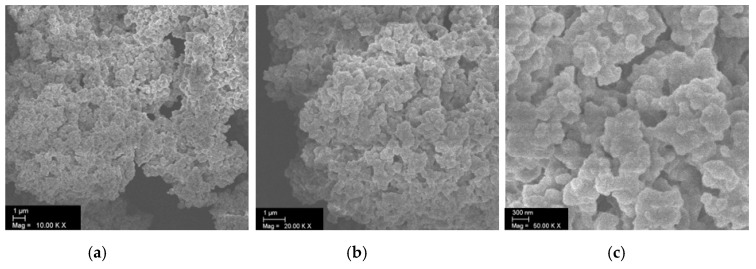
SEM image of the produced microclusters revealing pores of several microns. (**a**) 10,000× magnification, (**b**) 20,000× magnification, (**c**) 50,000× magnification.

**Figure 4:**
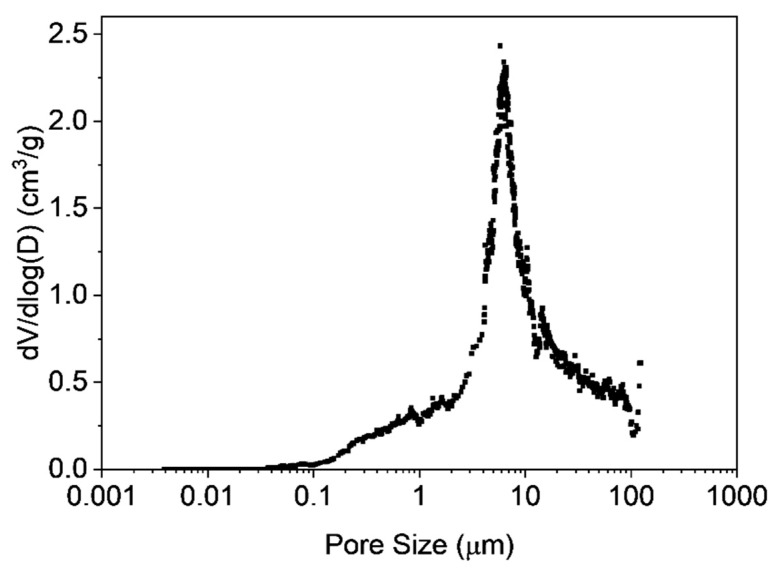
Differential pore size distribution of the microclusters by mercury intrusion experiments. The majority of the pores is in the order of several microns, whereas pores below 50 nm are virtually inexistent.

**Figure 5:**
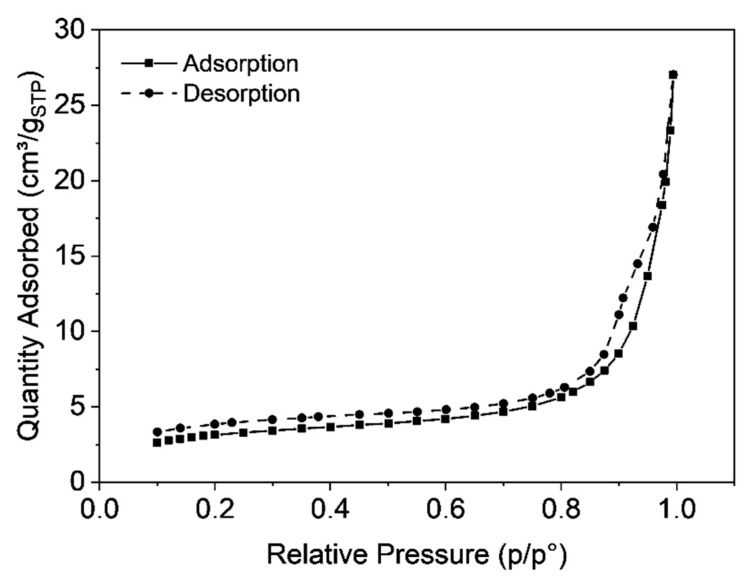
Nitrogen adsorption—desorption cycle giving a TYPE-II isotherm, which is characteristic for macroporous materials. The small hysteresis at higher relative pressures indicates capillary condensation.

Static light scattering was carried out to determine the size of the obtained microclusters. Figure 2 shows the particle size distribution with a mean value of 100 µm, whereas a fairly broad size distribution was observed. The SEM image (Figure 3) reveals the porous polymer scaffold of irregular shape exhibiting pore sizes in the micrometer range. It also shows that the primary nanoparticles clearly interpenetrated in each other’s shell. This is the result of the particular primary particle architecture having a soft-shell allowing interpenetration but a hard, highly cross-linked core, that hampers coalescence on the other hand. The pore size distribution was further investigated by mercury intrusion porosimetry as shown in Figure 4. It can be seen that the obtained porous microclusters exhibit a broad pore size distribution where the majority of pore sizes is in the range between 5 and 10 µm. Indeed, the shown specimen can be referred to as a macroporous material as virtually no pore sizes below 50 nm could be detected, indicating negligible presence of micro- or mesopores according to IUPAC nomenclature [33]. These findings are also in line with the results obtained from gas physisorption and the adsorption and desorption curves, which are shown in Figure 5. The obtained isotherm is a type-II isotherm, which is characteristic of macroporous materials according to the IUPAC classification [34]. This is due to unrestricted monolayer-multilayer adsorption, typical of physical adsorption on macroporous adsorbents [35]. The absolute surface was found to be only 11.4 m^2^/g as a consequence of the interpenetration of the primary nanoparticles, which clogs the majority of the smaller pores. However, there is a marginal hysteresis between the adsorption and desorption curves at higher relative pressures which is associated with the filling and emptying of mesopores by capillary condensation, confirming the minor relevance of mesopores. The asymptotic increase of the adsorbed nitrogen volume at relative pressure approaching one, indicates instead a significant population of macropores, though [31]. Since the adsorption in micropores occurs at lower relative pressures, the slope and height are indicators for micropores. In fact, the adsorbed volume at low relative pressures is very low (<5 cm^3^/g) which excludes the presence of micropores. An in-depth analysis of the tuning of product properties as cluster size, specific surface or pore-size distribution by alteration of the primary nanoparticle architecture is published elsewhere [25].

### 3.4. Functionalization by Click-Chemistry & Protein Immobilization 

**Figure 6:**
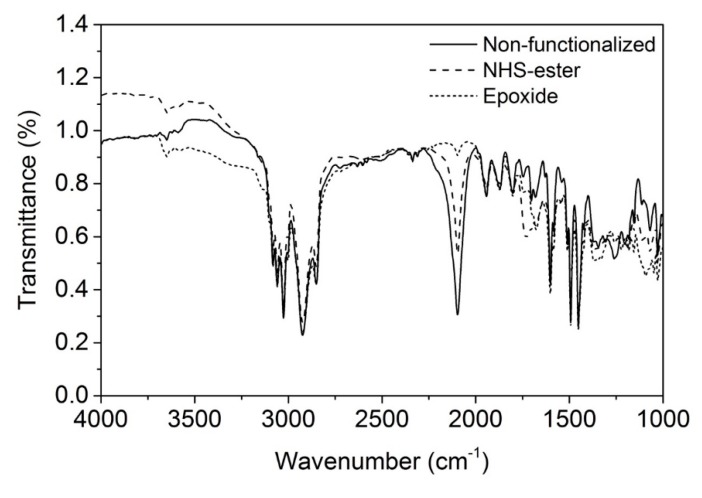
FT-IR spectrum of non-functionalized macroporous microclusters bearing azide on their surface (solid line) and of functionlized microclusters (dashed lines). The azide stretching band can be seen at 2100 cm^−1^.

The macroporous materials were chemically modified by click-chemistry to introduce active sites, which are commonly used for protein immobilization on the polymer surface. In a first experiment, propargyl-dPEG1-NHS-ester was attached to the polymer surface which can readily be attacked by nucleophiles present in proteins (e.g., primary amines from lysine). Figure 6 shows the FT-IR spectra of the pristine azide-containing starting material and its characteristic asymmetric azide stretch at 2100 cm^−1^. This specific stretch disappears at full conversion, after the 1,3-dipolar cycloaddition yielding a triazole ring. Since the aromatic C-H stretch remains unaffected by the cycloaddition, it can be used as internal reference for semi-quantitative yield determination. In fact, it can be seen that the azide band regressed but did not fully disappear upon the reaction with propargyl-dPEG1-NHS-ester (Figure 6), giving a yield of approximately 55%. Contrarily, the reaction with propargylglycidyl ether gave a yield of 98% (Figure 6). 

**Figure 7:**
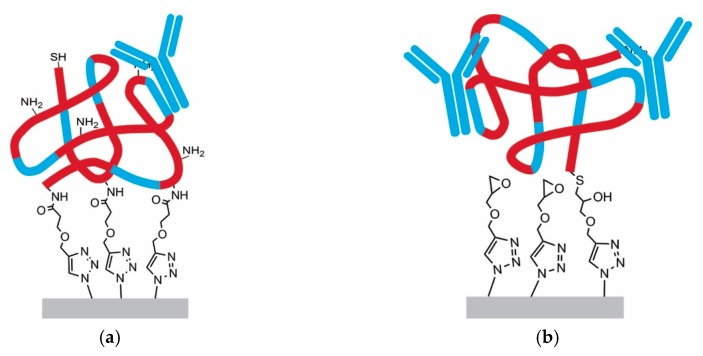
Illustrative comparison between multipoint attachment and single point attachment of proteins. (**a**) amidation reaction; (**b**) thiol-epoxide ring-opening reaction.

After the click reaction, *Staphylococcus aureus* protein A was immobilized as model protein as it is known to strongly interact with epitopes in the Fc-region of immunoglobulin G antibodies, and therefore relevant for industrial purification of these therapeutic proteins. Hence, protein A was attached to the polymeric scaffold by NHS-amidation or thiol epoxide ring-opening reaction. Two different protein A molecules were used, recombinantly produced protein A with the same primary structure as the native protein and a recombinant version with an introduced cysteine unit at the C-terminus exhibiting a thiol group that can be used for site-selective immobilization. The native-like protein A was linked to the NHS–ester material via multi-point attachment by exploiting the primary amines in protein A’s primary structure (1 N-terminus, 27 lysines). Contrarily, the recombinant protein A with the terminal cysteine undergoes a thiol-epoxide ring-opening reaction, which ideally is linked to the polymer via single-point attachment, as illustrated in Figure 7. 

Subsequently, static binding capacities towards monoclonal antibodies were determined. As expected, the single-point attachment using thiol-epoxide linkage revealed a three times higher static binding capacity (~15 mg/mL) towards IgGs compared to the multipoint attachment using the amidation reaction (~5 mg/mL). Since protein A is a globular molecule, most probably the amidation linkage resulted in different orientations, whereas for the thiol-epoxide reaction a uniform orientation can be assumed. Therefore, some protein binding epitopes of protein A are not accessible upon immobilization. The lower capacities can also be attributed to the fact that multipoint attachment leads to alterations in the tertiary structure of protein A and thus to a loss in its biological activity, a phenomenon that has often been reported [15,36].

### 3.5. Single-Point Attachment by Thiol-Epoxide Click Chemistry 

**Figure 8:**
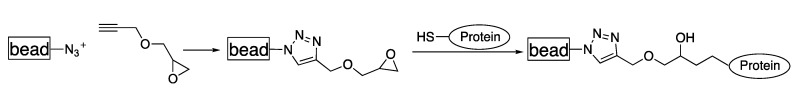
Chemical scheme for the chosen immobilization route starting from azide-containing microclusters.

As described in the previous section the preferred immobilization protocol is the single point attachment using thiol-epoxide ring-opening reactions (Figure 8) due to the proven higher binding capacities, but also due to easier handling as NHS-esters are susceptible to hydrolysis reactions. Table 4 summarizes the conducted reactions for the single-point attachment, where the impact of solvent is investigated on (i) the yield of the click reaction and (ii) the performance of obtained materials for antibody chromatography. For the reactions S-1 and S-2, the click reaction of propargylglycidyl ether to the base material was carried out prior to protein immobilization. On the other hand, the thiol-epoxide ring opening reaction was conducted first in reaction S-3, and the obtained molecule was clicked by CuAAC subsequently. As shown in Table 4, S-1 and S-2 reveal that DMF has a positive impact on the click yield, most probably because it swells the styrene-based polymer making the azide groups on the surface more accessible. Interestingly, the much higher yield in S-2 did not lead to higher IgG recovery, which remained substantially unchanged in the two cases. Apparently, reacting 10% (0.03 mmol_epoxide_/g_polymer_) of the available azide groups on the surface is already sufficient to immobilize the maximum amount of the bulky protein A molecule. This is coherent with the rather low BET surface of the material that is 11.4 m^2^/g, which strongly supports the hypothesis that only minor quantities of protein A can be attached in any case. S-3 was carried out in an aqueous buffer without any additional organic solvent. Similar to the previously described experiment, the yield for the click step is lower than the one in pure DMF. Given the fact that the yield of S-3 is the same as for S-1, one could expect a similarly high binding capacity: instead, the observed capacity was roughly 35% smaller. However, in this case, the thiol-epoxide reaction was carried out prior to the CuAAC reaction and likely more than one propargylglycidyl ether molecule reacted per protein A molecule, thus also resulting in some degree of multipoint attachment.

**Figure 9:**
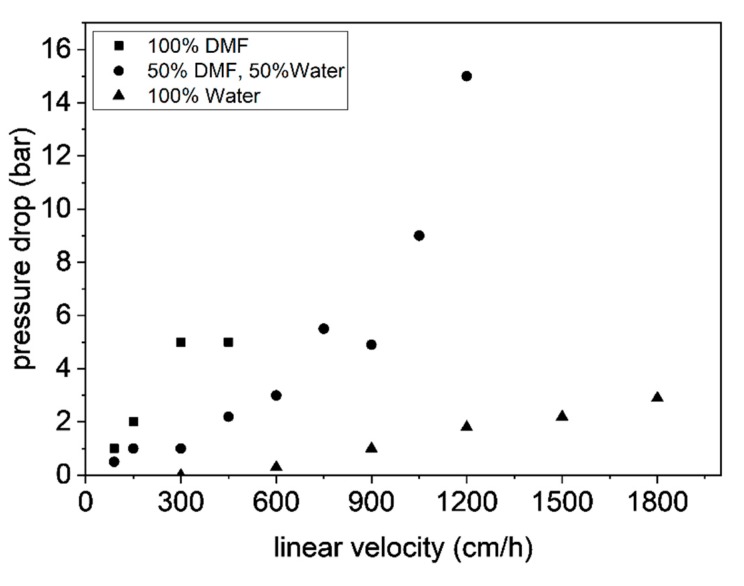
Impact of solvent used during the click reaction on pressure drop increase during aqueous phase chromatography at different linear velocities.

Despite the lower binding capacity, the material that was functionalized without organic solvent (S-3) has one crucial advantage regarding chromatographic applications. In particular, when packing the column with materials functionalized using DMF the observed increase in pressure drop with the flow rate was much higher compared to the DMF-free counterpart, as shown in Figure 9. As mentioned above, DMF swells the polymer, but protein purification is done in aqueous buffers, which in contrast results in shrinking the material. Even if the material was washed exhaustively after the click step, there is likely some DMF trapped in the polymer scaffold keeping parts of the material swollen. During the protein chromatography process, the material contracts leading to a rearrangement in the polymer three-dimensional structure, which clogs the column and consequently increases the pressure drop. 

**Figure 10:**
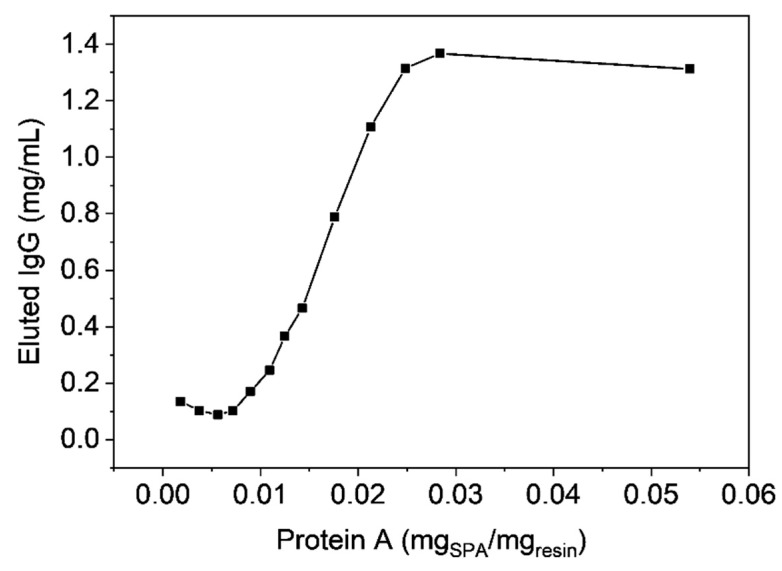
Static binding capacities determined for samples with different amounts of protein A per resin.

In the following experiment it was investigated how much protein A can be immobilized to the polymer by single point attachment, firstly by systematically adding different amounts of protein A to a constant amount of epoxide-bearing base material and then determining the static binding capacities, upon exhaustive washing to remove unbound proteins, by binding and eluting IgGs. As one can see from Figure 10, the maximum protein A loading is approx. 30 µg per mg polymer.

### 3.6. Antibody Chromatography 

**Figure 11:**
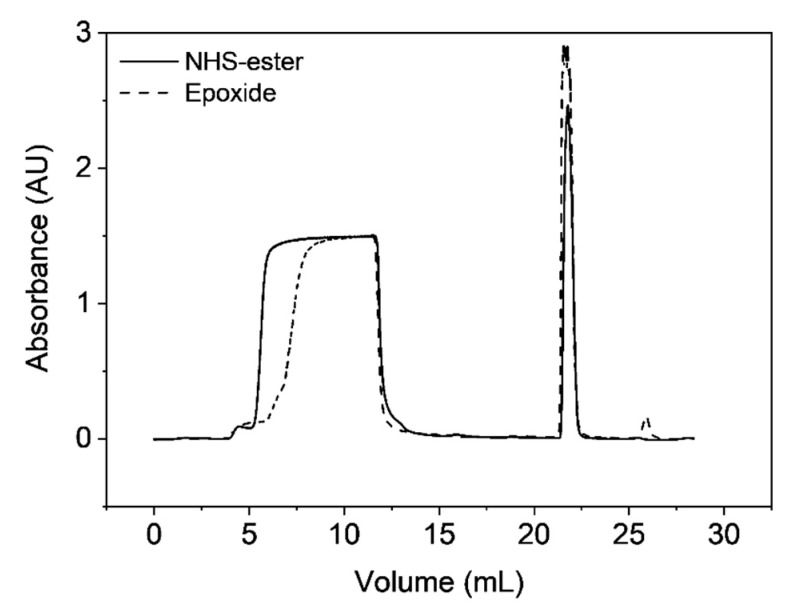
Breakthrough curves of produced protein A resins using microclusters with either NHS–ester or epoxide groups on their surface.

The obvious application of the obtained Protein A functionalized chromatographic materials is to bind monoclonal antibodies in the capture step of corresponding purification processes. In Figure 11 the breakthrough curves in the case of multi-point and single-point attachment are depicted, revealing a steep breakthrough curve, which indicates high capacity utilization. This appears to be much better than typically found for commercial chromatography resins used for antibody capture that show a shallow breakthrough curve, which results in product loss. This is related to the strongly diffusion-limited intra-particle mass transfer exhibited by these materials, which is instead strongly improved by the peculiar macroporous structure of the materials presented in this work. Nevertheless, the found dynamic binding capacities at 90 cm h^−1^ are only 12.7 mg mL^−1^ for the single point attachment and 6.1 mg mL^−1^ for the multipoint attachment, which can also be attributed to the low surface area of the produced materials. 

An in-depth chromatographic investigation, including dynamic binding capacities and comparisons with commercial chromatography resins, is reported elsewhere [37].

## 4. Conclusions

Herein, a solvent-free process, called reactive gelation, towards macroporos polymer–protein hybrid materials is presented. Using copper-mediated click chemistry, a ter-polymer based on styrene, divinyl benzene, and *4-*vinylbenzyl azide was modified to attain macroporous polymeric microclusters with active sites for protein-conjugation in form of epoxides or *N*-hydroxysuccinimide. In a subsequent step, *Staphylolloccous aureus* protein A was immobilized and the obtained protein-polymer hybrid materials were packed in a chromatographic column. As protein A is commonly used for downstream processing of monoclonal antibodies, the capture ability of the produced materials was investigated revealing that the single-point attachment gave up to three times higher static binding capacities than the multi-point coupling through amidation reactions. However, the highest static binding capacity of 15 mL mL^−1^ is still far below those of commercial products and requires further optimization. Nevertheless, it was shown that, due to their peculiar macroporous stucture, the developed materials show superior intraparticle mass transfer compared to the typical behavior of commercial protein A products, and, therefore, provide a promising alternative the purification in industrial processes. 

## Figures and Tables

**Table 1:** Recipe for the latex synthesis of produced polymer particles.

	Core Synthesis			Shell Synthesis	
	Initial Charge [g]	Initiator Shot [g]	Feed [g]	Feed [g]	Initiator Shot [g]
water	650.0	100.0	45.0		12.0
styrene			32.0	17.3	
DVB			8.0	0.6	
VBA				11.3	
SDS	4.1		1.3		
KPS		1.5			0.3

**Table 2:** Overview of Diameter, Polydispersity Index (PDI), conversion and dry content during the emulsion polymerization.

Time [min]	Diameter [nm]	PDI	Conversion [%]	Dry Content [%]
60	24	0.12	97	2.5
120	27	0.06	98	4
180	30	0.03	99	5.5
240	31	0.06	99	6.5
300	34	0.04	97	7.4
360	38	0.06	99	8.6

**Table 3:** Elemental analysis of the produced macroporous base material.

C	H	N
[%]	[%]	[%]
84.2	7.5	3.4

**Table 4:** Overview of conducted reactions yielding functional materials with covalently bounded staphylococcus aureus protein A.

	**Reaction 1**		**Reaction 2**	
**Click Reaction (CuAAC)**			**Thiol-Epoxide Ring Opening Reaction**	
**S-1**	50% DMF 50% Water	10% yield	Aq. Buffer pH 7.5	15.2 mg_IgG_/mL_polymer_
**S-2**	100% DMF	98% yield	Aq. Buffer pH 7.5	14.3 mg_IgG_/mL_polymer_
**Thiol-Epoxide Ring Opening Reaction**			**Click Reactions (CuAAC)**	
**S-3**	Aq. Buffer pH 7.5	9.6 mg_IgG_/mL_polymer_	Aq. Buffer pH 7.5	10% yield
